# Endothelin-1 enhances fibrogenic gene expression, but does not promote DNA synthesis or apoptosis in hepatic stellate cells

**DOI:** 10.1186/1476-5926-5-5

**Published:** 2006-10-24

**Authors:** Masahiko Koda, Michael Bauer, Anja Krebs, Eckhart G Hahn, Detlef Schuppan, Yoshikazu Murawaki

**Affiliations:** 1 First Department of Medicine, University of Erlangen-Nuernberg, Erlangen, Germany; 2 Second Department of Internal Medicine, Faculty of Medicine, Tottori University, Yonago 683-8504, Japan; 3 Division of Gastroenterology, Beth Israel Deaconess Medical Center, Harvard Medical School, Boston, MA, USA

## Abstract

**Background:**

In liver injury, the pool of hepatic stellate cell (HSC) increases and produces extracellular matrix proteins, decreasing during the resolution of fibrosis. The profibrogenic role of endothelin-1 (ET-1) in liver fibrosis remains disputed. We therefore studied the effect of ET-1 on proliferation, apoptosis and profibrogenic gene expression of HSCs.

**Results:**

First passage HSC predominantly expressed endothelin A receptor (ETAR) mRNA and 4th passage HSC predominantly expressed the endothelin B receptor (ETBR) mRNA. ET-1 had no effect on DNA synthesis in 1st passage HSC, but reduced DNA synthesis in 4th passage HSC by more than 50%. Inhibition of proliferation by endothelin-1 was abrogated by ETBR specific antagonist BQ788, indicating a prominent role of ETBR in growth inhibition. ET-1 did not prevent apoptosis induced by serum deprivation or Fas ligand in 1st or 4th passage HSC. However, ET-1 increased procollagen α1(I), transforming growth factor β-1 and matrix metalloproteinase (MMP)-2 mRNA transcripts in a concentration-dependent manner in 1st, but not in 4th passage HSC. Profibrogenic gene expression was abrogated by ETAR antagonist BQ123. Both BQ123 and BQ788 attenuated the increase of MMP-2 expression by ET-1.

**Conclusion:**

We show that ET-1 stimulates fibrogenic gene expression for 1st passage HSC and it inhibits HSC proliferation for 4th passage HSC. These data indicate the profibrogenic and antifibrogenic action of ET-1 for HSC are involved in the process of liver fibrosis.

## Background

Hepatic stellate cells (HSC) are responsible for the storage of retinoid and the control of sinusoidal blood flow in normal liver. In liver injury, HSC number is markedly increased and transformed into myofibroblast-like cells, termed activated HSC. Activated HSC produce extracellular matrix components, matrix metalloproteinases and their inhibitors [1-3]. All of them decreasing during the resolution of the fibrotic tissue.

Endothelin (ET)-1, a 21 amino acid peptide, plays multifunctional roles in a variety of tissues and cells [4,5]. In the liver, ET-1 induces vascular constriction and stimulates glycogenolysis and the synthesis of lipid mediators [6,7]. ET-1 is secreted by sinusoidal endothelial cells and by activated HSC [8], and activated HSC that express high numbers of ET receptors [1] respond to ET-1 with spreading and expression of α-smooth muscle actin [8,9]. The cellular receptors for ET-1 are the endothelin A receptor (ETAR) and the endothelin B receptor (ETBR) [10,11]. The expression of ETAR and ETBR are different between quiescent and activated HSC or between early- and late-activated states in HSC.

ET-1 is involved in the evolution of tissue fibrosis and ET-1 overexpressing transgenic mice develop renal fibrosis [12]. ET-1 can increase collagen synthesis in cardiac fibroblasts and vascular smooth muscle cells [13,14]. In the liver ET-1 contributes to HSC activation and fibrogenesis by upregulation of type I collagen gene expression [15]. We previously showed that in a rat model of secondary biliary fibrosis a selective ETAR antagonist reduced collagen accumulation even in an advanced stage of fibrosis [16]. However, the exact role of ET-1 as a modulator of HSC proliferation, apoptosis and extracellular matrix metabolism remains unclear. Therefore, in the present study we investigated the effects of ET-1, as well as the ETAR and the ETBR on the proliferation, apoptosis and extracellular matrix production of HSC in states of early and late activation, corresponding to different expressions of ETAR and ETBR.

## Results

### The gene expression of ETAR and ETBR in 1st or 4th passage HSC

**Figure 1:**
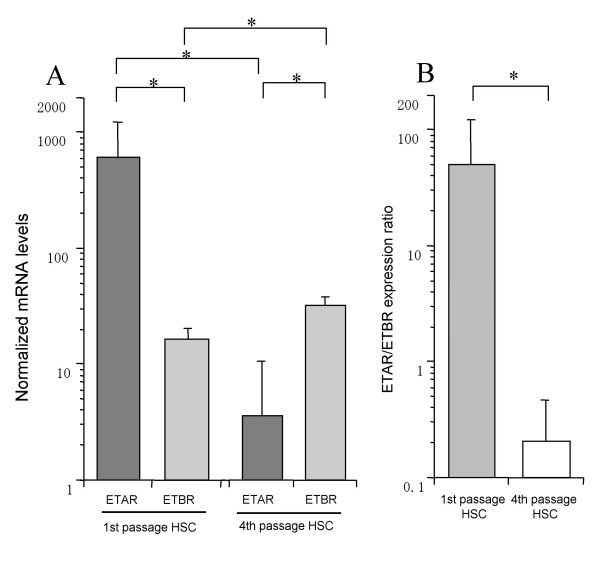
**The gene expression of ETAR and ETBR in early- and late-passage HSC**. **A**: The gene expression of ETAR and ETBR in 1st and 4th passage HSC. **B**: the relative expression ratio of ETAR to ETBR in 1st and 4th passage HSC. HSC were plated on 25 cm^2 ^dishes at a density of 1.0 × 10^5 ^cells/dish in DMEM containing 10% FCS. After confluence, cells were washed with PBS and placed in DMEM with 0.125% FCS for 24 hours. RNA isolation and real time PCR using SYBR Green were performed according to Material and Methods. Data were normalized to GAPDH mRNA levels. Results are given as mean ± SD (n = 5). *: p < 0.05.

In 1st passage HSC, the ETAR mRNA expression was significantly higher than the ETBR mRNA expression (Fig. 1). However, the ETAR mRNA dramatically decreased in 4th passage HSC. On the other hand, the ETBR mRNA expression significantly increased 1.9-fold in 4th passage HSC. The relative expression ratio of ETAR to ETBR was higher in 1st passage HSC than in 4th passage HSC.

### Effect of ET-1 on HSC proliferation

**Figure 2:**
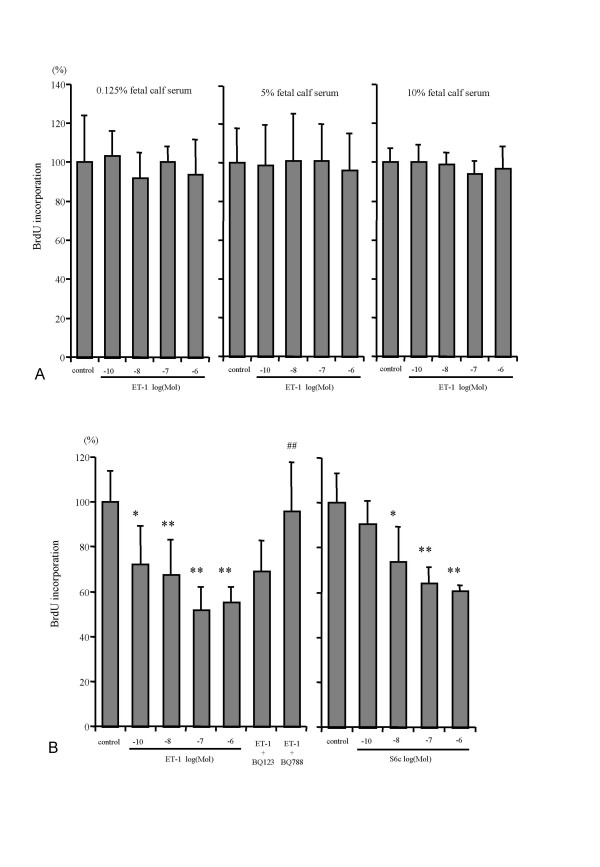
**Effect of ET-1 on DNA synthesis and proliferation in HSC**. **A: **Effect of ET-1 on DNA synthesis of 1st passage HSC. BrdU incorporation into DNA was measured in 8 × 10^3 ^HSC during 48 h and at different concentrations of FCS. Absorbance values of controls were set as 100% for each concentration of FCS and values normalized to the respective control culture levels. Results were means ± SD (n = 8). There was no effect of ET-1 on the proliferation of 1st passage HSC. **B: **Inhibitory effect of ET-1 on the proliferation of 4th passage HSC. BrdU incorporation into DNA was measured in 8x10^3 ^HSC during 48 hours at different concentrations of ET-1 or the ETBR agonist S6c with 10% FCS. The ETAR antagonist, BQ123, or the ETBR antagonists, BQ788, were added at 10^-6 ^M in the presence of 10^-6 ^M of ET-1. Results are given as mean ± SD (n = 8). Absorbance values of controls were set as 100% and values for cultures under the influence of each concentration of ET-1 or S6c are shown. *p < 0.05, **p < 0.01 vs control, ## p < 0.01 vs ET -1 10^-6 ^M.

ET-1 in 1st passage HSC did not affect DNA synthesis in the presence of 0.125%, 5% or 10% fetal calf serum (FCS) (Fig. 2A), or in the presence of 10^-6 ^M of BQ123, a selective ETAR antagonist, or BQ788, a selective ETBR antagonist (data not shown). In contrast, ET-1 (10^-10^, 10^-8^, 10^-6 ^M) dose-dependently reduced DNA synthesis of 4th passage HSC only in 10% FCS, with maximal inhibition (49.3%) at 10^-7 ^M ET-1 (Fig. 2B). This effect was mediated by the ETBR, since Sarafotoxin (S6c), a selective ETBR agonist, dose-dependently inhibited DNA synthesis (40% inhibition at 10^-6 ^M), even in the absence of ET-1 (Fig. 2B). The involvement of the ETBR was confirmed when ET-1 (10^-6 ^M) in the presence of the ETAR antagonist BQ123 (10^-6 ^M) still reduced DNA synthesis, while the combination of ET-1 and the ETBR antagonist BQ788 (10^-6 ^M) abrogated the inhibitory effect of ET-1 on serum-stimulated DNA synthesis.

### Effect of ET-1 on HSC apoptosis

**Figure 3:**
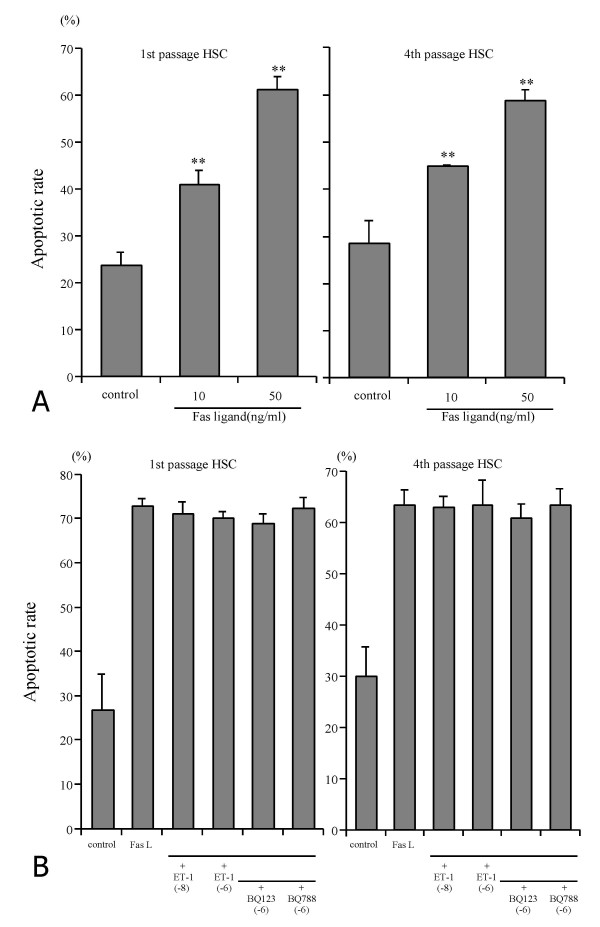
**HSC apoptosis**. **A **:Apoptotic rate of 1st and 4th passage HSC induced by different concentrations of Fas-ligand. Apoptotic rate of HSC was measured after HSC were incubated in DMEM with 0.125% FCS containing Fas-ligand at different concentrations for 24 hours. Results are given as mean ± SD (n = 4). **: p < 0.01 vs control. **B **:Effect of ET-1 and ET receptor antagonists on Fas-induced apoptosis of 1st and 4th passage HSC. HSC in DMEM containing 0.125% FCS were incubated with ET -1 alone at 10^-8 ^M or 10^-6 ^M with or without Fas-ligand (50 ng/ml), or with ET-1 (10^-6 ^M), FasL and the ETAR antagonist BQ123 or the ETBR antagonist BQ788 (both at 10^-6 ^M). Results are given as mean ± SD (n = 8).

Spontaneous apoptosis rate was 0.99 ± 0.08% (mean ± SD, n = 6) in 1st and 2.86 ± 0.52% in 4th passage HSC (n = 6) when cultured in 10% FCS for 24 h. Addition of ET-1 (10^-10^, 10^-8^, 10^-6 ^M) did not alter the basal level of apoptosis (1.03%, 1.32% and 1.19%, respectively, in 1st passage HSC, and 1.46%, 1.53% and 1.25%, respectively in 4th passage HSC). To induce significant apoptosis, cells were either serum-deprived or treated with Fas-ligand (Table 1, Fig. 3A). ET-1 (10^-8 ^M or 10^-6 ^M) had no effect on apoptosis induced by serum deprivation in early and late passage HSC, and did not rescue the cells from apoptosis when added one h before addition of Fas-ligand (Fig. 3B). Simultaneous addition of ET-1 and the ETAR and ETBR antagonists also did not alter Fas-ligand induced apoptosis both in 1st and 4th passage HSC.

**Table 1:** The effects of endothelin-1 on apoptosis induced serum deprivation in 1st and 4th passage HSC.

	1^st ^passage HSC			4^th ^passage HSC		
		
	Apoptosis after serum deprivation			Apoptosis after serum deprivation		
		
	72 hr (%)	120 hr (%)	168 hr (%)	72 hr (%)	120 hr (%)	168 hr (%)
Control	38.8 ± 7.5	40.2 ± 6.6	55.6 ± 10.0	40.2 ± 3.2	46.9 ± 5.2	42.3 ± 5.6
ET-1(10^-8 ^M)	37.3 ± 9.3	42.4 ± 4.6	56.3 ± 8.0	35.3 ± 4.4	46.9 ± 3.4	36.7 ± 3.3
ET-1(10^-6 ^M)	31.9 ± 9.9	40.1 ± 5.5	53.3 ± 11.3	36.7 ± 5.8	55.4 ± 14.3	46.2 ± 11.9

Data are given as mean ± standard deviation. ET-1: endothelin-1.

### Effect of ET-1 on HSC matrix-related gene expression

**Figure 4:**
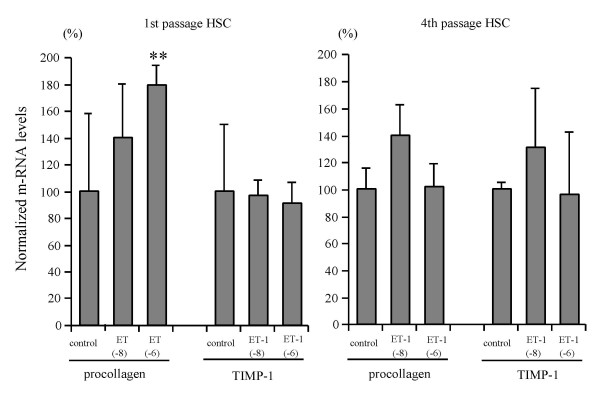
**Effect of ET-1 on procollagen type I and TIMP-1 transcript levels of 1st and 4th passage HSC**. Cells were incubated without or with 10^-8 ^M or 10^-6 ^M ET-1 for 48 hours. Total RNA from HSC was reverse transcribed and transcript levels of procollagen α1(I) and TIMP-1 were determined by real time quantitative PCR based on the Taqman technology. Data were normalized to GAPDH mRNA levels. Results are given as mean ± SD (n = 4). **: p < 0.01 vs controls.

**Figure 5:**
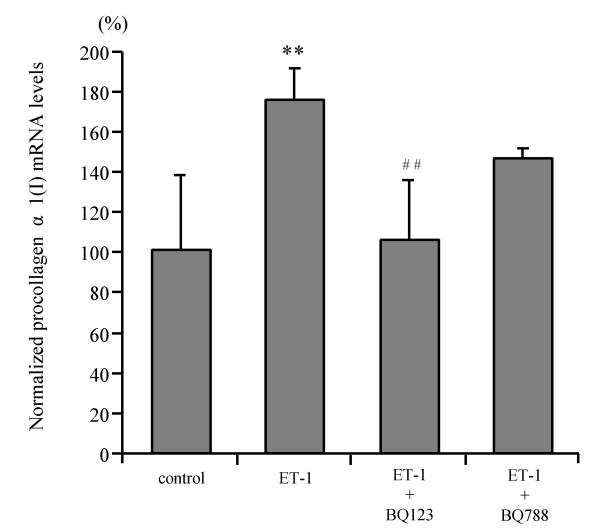
**Effect of ET receptor antagonists on procollagen α1(I) mRNA expression of 1st passage HSC**. Cells were stimulated with 10^-6 ^M ET-1 in the absence or presence of the ETAR antagonist BQ123 (10^-6 ^M) or the ETBR antagonist BQ788 (10^-6 ^M) for 48 hours. The mRNA levels were determined by real time quantitative PCR. Results are given as mean ± SD (n = 4). **p < 0.01 vs control, ^## ^p < 0.01 vs ET-1 (10^-6 ^M).

**Figure 6:**
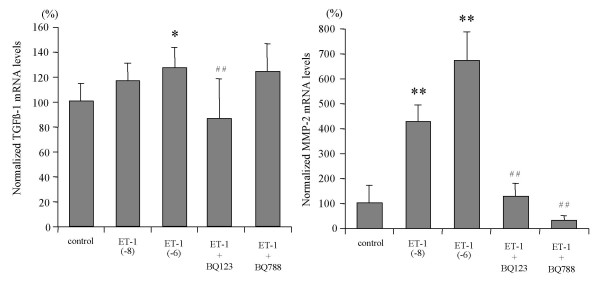
**Effect of ET-1 on TGFβ-1 and MMP-2 transcript levels of 1st passage HSC and influence of the ET receptor antagonists**. Cells were stimulated with 10^-6 ^M ET-1 in absence or presence of the ETAR antagonist BQ123 (10^-6 ^M) or the ETBR antagonist BQ788 (10^-6 ^M). TGFβ-1 and MMP-2 transcript levels were determined by real time quantitative PCR, using Taqman technology, and data were normalized to GAPDH mRNA. Results are given as mean ± SD (n = 4). **: p < 0.01, *: p < 0.05 vs controls, ^## ^p < 0.01 vs ET-1 (10^-6 ^M).

ET-1 at concentrations of 10^-8 ^M and 10^-6 ^M increased procollagen α1(I) mRNA expression 1.4- and 1.8-fold, respectively, in 1st passage HSC, while no effect was found in 4th passage HSC (Fig. 4). Tissue inhibitor of metalloproteinase-1 (TIMP-1) transcript levels remained unchanged both in 1st and 4th passage HSC (Fig. 4). Only the ETAR antagonist, BQ123, completely blocked ET-1 enhanced procollagen α1(I) mRNA expression, while the ETBR antagonist, BQ788, had no effect (Fig. 5). ET-1 (10^-8 ^M and 10^-6 ^M) increased transforming growth factor β-1 (TGFβ-1) mRNA expression 1.2–1.3-fold in 1st passage HSC which was blocked by the ETAR antagonist. In addition, ET-1 (10^-8 ^M and 10^-6 ^M) upregulated matrix metalloproteinase-2 (MMP-2) mRNA transcripts 4- and 6-fold, respectively, in 1st passage HSC, and both the ETAR and the ETBR antagonist inhibited this induction completely (Fig. 6). In 4th passage HSC no effect of ET-1 on TGFβ-1 and MMP-2 mRNA expression was found (data not shown). [These findings clearly show that ET-1 stimulated profibrogenic gene expression, i.e. procollagen α1(I), TGFβ-1 and MMP-2 mRNA, only in 1st passage HSC and via the ETAR.]

## Discussion

Several studies have implicated ET-1 in fibrogenesis of the kidneys, the cardiovascular system and liver fibrosis. However, the role of ET-1 in hepatic fibrogenesis and in particular in HSC matrix production and apoptosis remains controversial. Therefore we examined cell proliferation, apoptosis and extracellular matrix metabolism of ET-treated HSC in an early and a late state of activation. We used 1st passage and 4th passage HSC as an early and late state of activation. Our study has shown that ETAR is dominant in 1st passage HSC and ETBR is dominant in 4th passage HSC. Our results agreed with those reported by other investigators [11,20]. The progressive activation in HSC in culture is associated with progressive shift from a relative predominance of ETAR to relative predominance of ETBR. A predominance of ETBR was observed when the cells had undergone complete transition to myofibroblastic-like phenotype. In vivo study, ETBR is predominantly expressed in both normal liver and cirrhotic liver and overexpressed especially in cirrhotic liver [21,22]. Taken together, HSC predominantly express ETAR in early state, activated by several cytokine or liver damage, and predominantly express ETBR on late activated state.

Previous studies reported the mitogenic potential of ET-1 in coronary smooth muscle cells and alveolar fibroblasts [23,24], and Rockey et al. [8] and Pinzani et al. [11] demonstrated that ET-1 stimulates DNA synthesis in early cultured HSC in the presence of low concentrations of FCS. We were unable to demonstrate any mitogenic effect of ET-1 in 1st and 4th passage HSC. Moreover, we found inhibition of cell proliferation by ET-1 in 4th passage HSC. This discrepancy is likely explained by primary culture and low FCS concentrations (2–5%) which those authors used, whereas we used activated HSC after one or 4th passages that appear to resemble the myofibroblasts obtained by outgrowth from explants of normal liver by Mallat et al. [20]. In these myofibroblastic HSC use of the selective ETBR agonist sarafotoxin (6Sc) and the selective ETBR antagonist (BQ788) demonstrated that this growth inhibitory effect was mediated by the ETBR. We showed that passaging of HSC induced a predominance of ETBR over the ETAR. Taken together, we conclude that ET-1 induces inhibition of cell proliferation in long-term activated but not early HSC, and that ET-1 does not contribute to liver fibrosis due to stimulation of HSC proliferation.

Although ET-1 has been described as a survival factor for various kinds of cells [25,26], its effect on HSC apoptosis had not been studied. Using serum deprivation we were able to induce a reproducible apoptosis rate of 40% both in 1st and 4th passage HSC. In addition, we induced HSC apoptosis via the Fas signaling cascade. Fas has been demonstrated to be expressed in liver and to be overexpressed in acute or chronic liver diseases [27,28]. Furthermore, activated HSC are more susceptible to Fas-ligand induced apoptosis than quiescent HSC [19,29-31]. Using both proapoptotic stimuli, ET-1 did not rescue 1st or 4th passage HSC from apoptosis.

We could show that ET-1 dose-dependently stimulated the expression of procollagen α1(I) mRNA in 1st, but not in 4th passage HSC. Similarly, ET-1 upregulated the expression of TGFβ-1, the strongest profibrogenic cytokine. These fibrogenic functions of ET-1 were inhibited by the ETAR antagonist. Our findings are in accord with data showing that early passage HSC predominantly express the ETAR, whereas 4th passage HSC and myofibroblasts obtained by outgrowth mainly express the ETBR [11,20]. Contrary to our results, Gandhi et al. [32] reported that ET-1 stimulates collagen synthesis in HSC via the ETBR. Although the cause of this difference is unknown, many reports have shown that the stimulatory effect of ET-1 for procollagen synthesis in fibroblasts and vascular smooth muscle cell is mediated by the ETAR [13,14], in agreement with our present findings in HSC. Furthermore, we could demonstrate [16] that only an ETAR in contrast to a mixed (ETAR and ETBR) antagonist [33] inhibits hepatic fibrosis in rats with secondary biliary fibrosis due to bile duct ligation and scission in vivo.

Excess extracellular matrix proteins are degraded by matrix metalloproteinases (MMPs), which are regulated by specific inhibitors, in particular tissue inhibitor of MMPs 1 (TIMP-1), which appears to play an important profibrogenic role in hepatic fibrogenesis [34]. We found no effect of ET-1 on TIMP-1 expression in 1st and 4th passage HSC. However, ET-1 stimulated the expression of MMP-2 mRNA in 1st passage HSC. The upregulation of MMP-2 favours degradation of the normal subendothelial matrix, with subsequent replacement by a nonfunctional interstitial extracellular matrix, including procollagen I. It also accelerates HSC activation and invasiveness [35,36]. Therefore, ET-1 further likely promotes unfavourable matrix turnover through the stimulation of collagen 1, TGFβ-1 and MMP-2.

Although procollagen α1(I) or TGFβ-1 expression were suppressed only by the ETAR antagonist, MMP-2 expression induced by ET-1 was inhibited both by the ETAR and the ETBR antagonist. The reason for this is yet unclear. It can be speculated that the regulation of MMP-2 expression may involve other promoter elements than those stimulated by TGFβ-1. Thus the NFκB family of transcription factors induces expression and activation of MMP-2 [37]. Furthermore, ET-1 enhances the DNA-binding activity of NFκB via ETBR [38]. Therefore, MMP-2 may be upregulated by both ET-receptors via NFκB.

While it is still not possible to examine to which stages of liver fibrosis progression early and late passage HSC correspond, hepatic concentrations of ET-1 and densities of ET-receptors are increased in human and experimental liver cirrhosis [8,11,32], part of which are contributed by sinusoidal endothelial cells [39]. Interestingly, a recent report has shown that TGFβ-1 reduces ET receptor density in HSC, especially that of the ETBR [40]. Our observation that 4th passage HSC express more functional ETBR than ETAR are in line with findings that 4th passage HSC become less sensitive to auto- and paracrine TGFβ-1 stimulation [41].

## Conclusion

ET-1 stimulates the expression of procollagen α1(I) and TGFβ-1 (through the ETAR) and MMP-2 (through both ETAR and ETBR) in 1st passage HSC, whereas it inhibits HSC proliferation in late stages of HSC activation. This suggests that ET-1 is profibrogenic in early and possibly antifibrogenic in late stages of hepatic fibrogenesis.

## Materials and methods

### Materials

Cell culture materials were purchased from Biochem AG (Berlin, Germany) or Life Technology (Karlsruhe, Germany). ET-1, the ETBR agonist sarafotoxin S6c, the ETAR antagonist BQ123, the ETBR antagonist BQ788. Fas ligand were purchased from Alexis Biochemicals (Gruenburg, Germany). BrdU colorimetric cell proliferation ELISA was from Roche (Mannheim, Germany). Primers and probes for real time PCR were synthesized at MWG-Biotech AG (Ebersberg, Germany) and Superscript II RNase H^- ^reverse transcriptase was from Life Technologies (Karlsruhe, Germany). Random hexamers and oligo(dT) primer were from Promega (Mannheim, Germany). Other reagents were purchased from Sigma (Seele, Germany).

### Cell preparation

HSC were isolated from male Wistar rats (400–500 g, from Schoenwalde, Germany) fed ad libitum using the collagenase-perfusion method and purified on a Nycodenz gradient as described (17). In brief, the liver was perfused through the portal vein and using an inferior vena cava outflow using calcium free Hank's balanced salt solution (HBSS) (Life Technology, Karlsruhe, Germany) maintained at 37°C at a rate of 10 ml/min for 10 min. The perfusion was continued with HBSS containing 1.3 mM CaCl_2_, 0.08% protease E, 0.05% collagenase type IV and 0.001% DNase 1 at a rate of 10 ml/min for 30 min. The cell suspension was subjected to density gradient centrifugation. The HSC-enriched fraction was suspended in DMEM containing penicillin (250 U/ml), streptomycin (250 μg/ml) and 10% FCS, and seeded at a density of 1 × 10^6 ^cells/ml. Cell viability was greater than 91% as determined by Trypan Blue exclusion. HSC purity, as assessed by phase-contrast microscopy and vitamin A autofluorescence immediately after plating, and by immunoreactivity for desmin one week after plating, was greater than 95%, with a yield ranging from 1.2 × 10^7 ^to 1.5 × 10^7 ^HSC/rat. The cells were subcultured (split ratio 1:3) in DMEM with 10% FCS, penicillin (100 IU/ml), streptomycin (100 μg/ml) and amphotericin B.

We used 1st passage HSC as an early activated state and 4th passage HSC as a late activated state.

### DNA synthesis

Cells were plated in 96-well dishes at a density 8 × 10^3 ^cells/well in complete culture medium. After 24 hours the cells were washed with PBS and placed in DMEM with 0.125% FCS for 48 hours. This medium was removed and the cells were placed in fresh DMEM with 0.125%, 2% or 10% FCS containing ET-1 at different concentrations. After 48 hours incubation with BrdU at 37°C, BrdU incorporated into DNA was measured by ELISA according to the manufacture's protocol.

### Induction of apoptosis

Either serum deprivation [18] or Fas-ligand [19] were used to induce apoptosis in HSC. In serum deprivation apoptosis, control was made with 10% serum. In Fas-ligand induced apoptosis, control was run without Fas-ligand. There was no vehicle control. For serum deprivation, the cells were plated in 6-well dishes at a density of 2 × 10^4^/well in complete culture medium. After 24 h the cells were washed with PBS and placed in DMEM with 0.125% FCS containing ET-1 at increasing concentrations for 72, 120 or 168 h. The medium was exchanged after 72 or 120 h and the apoptotic rate measured by flow cytometry. For Fas-ligand induced apoptosis, the cells were seeded in 6-well dishes in complete culture medium and placed in DMEM with 0.125% FCS for 24 h as before. After 24 h the medium was replaced by fresh DMEM with 0.125% FCS containing 1 μg/ml of Fas enhancer (mouse IgG) and 10 to 50 μg/ml Fas-ligand for 24 h. To investigate the influence of ET-1 on Fas-ligand induced apoptosis, HSC were cultured with increasing concentrations of ET-1 described above.

### Flow cytometric quantification of apoptotic HSC

HSC were trypsinized and centrifuged for 10 minutes at 500 g. Cells were fixed in 3 ml of 75% ethanol/25% PBS, diluted in 10 ml PBS and centrifuged for 10 minutes at 500 g. After resuspension in PBS, digestion of RNA with RNase A (500 μl of 500 μg/ml) at 37°C for 30 minutes and staining with propidium Iodide at a final concentration of 100 μg/ml, cell cycle stages were determined by flow cytometry (Coulter, Epics X/XL Flow cytometry System, Krefeld, Germany). SubG1 events were quantified as correlate for the rate of apoptosis. At least 12000 events were collected for each analyzed sample.

### Inhibition of ETA and ETB receptors

HSC were plated on 25 cm^2 ^dishes at a density of 1.0 × 10^5 ^cells/dish in DMEM containing 10% FCS. After confluence, cells were washed with PBS and placed in DMEM with 0.125% FCS for 48 hours. Thereafter cells were incubated with ET-1 for 48 hours in the presence of the ETAR antagonist BQ123 (10^-6 ^M) or ETBR antagonist BQ788 (10^-6 ^M).

### RNA isolation and reverse transcription

Total RNA of HSC was extracted by using the acid-phenol guanidium method. The RNA concentration was determined by absorbance at 260 nm and the RNA quality verified by electrophoresis on an ethidium bromide stained 1% agarose gel. Total RNA was reverse transcribed in a final volume of 20 μl containing 1 × RT buffer (500 μM each dNTP, 3 mM MgCl2 75 mM KCl, 50 mM Tris-HCl pH 8.3), 10 units of Superscript II RNase H^- ^reverse transcriptase (Gibco BRL, Life Technologies, Karlsruhe, Germany), 1 μl of 50 ng/μl random hexamers (Promega, Mannheim, Germany), 0.5 μl of 100 pmol/ml oligo(dT) primer and 1~5 μg of total RNA. The samples were incubated at 20°C for 10 minutes, 42°C for 30 min and reverse transcriptase was inactivated by heating to 99°C for 5 min and cooling to 5°C for 5 min.

### Real time quantitative PCR

We used a Light Cycler System (Roche, Tokyo) and a Light Cycler-FastStart DNA Master SYBR Green I kit to quantify mRNA of ETAR and ETBR. Nucleotide sequences of ETAR and ETBR for the primers were as follows; ETAR (accession no. NM012550) sense: -ACCAGTCCAAAAGCCTCA-, antisense: -TCTGCACAGGGTTAGTTCA-; ETBR (accession no. NM017333) sense: -AACTTCCGCTCCAGCAAT-, antisense: -TCCCGAGGCTTCATTCAT-. Conditions for real-time PCR were as follows: 10 min denaturing at 95°C, 10 s annealing at 64 or 62°C, and 5–9 s amplification at 72°C. Forty cycles were performed and then followed by melting curve analysis to verify the correctness of the amplification. Analysis of the data was performed according to the manufacturer's instructions, using Light Cycler software version 3.5.3.

The Taqman technology was used to quantify procollagen I, TIMP-1, TGFβ-1, and MMP-2 mRNA. This method relies on the correlation between the abundance of mRNA and the number of PCR cycles necessary to reach a threshold of detection of a fluorescent probe released during each successive replication. A standard curve performed with a serial dilution of a sample showed a constant slope when amplification occurred between 10 and 40 cycles. Real time quantitative PCR analysis was performed with a PE applied Biosystems 7700 sequence Detector (Perkin-Elmer Applied Biosystems, Faster City, CA), which is a combined thermal cycler and fluorescence detector. Specific primers and probes for real time PCR were chosen with the assistance of the software Primer Express (Perkin-Elmer Applied Biosystems, Faster City, CA). Rat nucleotide sequences for the primers and hybridization probes were as follows; glyceraldehyde 3-phosphate dehydrogenase (GAPDH)(accession no. M17701) sense: -CCT GCC AAG TAT GAT GAC ATC AAG A-, antisense: -GTA GGC CAG GAT GCC CTT TAG T-, probe: -CTC GGC CGC CTG CTT CAC CA-; procollagen I (α1) (accession no. Z78279) sense: -TTC GGC TCC TGC TCC TCT TA-, antisense: -GTA TGC AGC TGA CTT CAG GGA TGT-, probe: -TTC TTG GCC ATG CGT CAG GAG GG-; TIMP-1 (accession no. U06179) sense: -TCC TCT TGT TGC TAT CAT TGA TAG CTT-, antisense: -CGC TGG TAT AAG GTG GTC TCG AT-, probe: -TTC TGC AAC TCG GAC CTG GTT ATA AGG-; TGFβ-1 (accession no. X52498) sense: -AGAAGTCACCCGCGTGCTAA-, antisense: -TCCCGAATGCTCGACGTATTGA-, probe: -ACCGCAACAACGCAATCTATGACAAAACCA-; MMP-2 (accession no. X71466) sense: -CCGAGGACTATGACCGGGATAA-, antisense: CTTGTTGCCCAGGAAAGTGAAG-, probe: -TCTGCCCCGAGACCGCTATGTCCA-.

Ten microliters of the RT samples was used for quantitative two step PCR with a 5 minute denaturation step at 95°C, followed by 40 cycles of 15 seconds at 95°C and 1 min at 65°C in the presence of 200 nM specific forward and reverse primers, 100 mM specific fluorogenic probe, 5 mM MgCl2, 50 mM KCl, 10 mM Tris buffer (PH 8.3), 200 μM each dNTP, and 1.25 units of DNA polymerase. Each sample was analyzed in duplicate and a calibration curve constructed using a 2-fold serial dilution of a standard cDNA preparation obtained from total RNA of untreated HSC run in parallel with each analysis. For each sample, the amounts of procollagen α1(I), TIMP-1, TGFβ-1 and MMP-2 were divided by the amount of GAPDH to obtain normalized procollagen α1(I), TIMP-1, TGFβ-1 or MMP-2 values.

### Statistical analysis

Statistical analyses were performed using Statview Version 5 (SAS Institute Inc. NC). Significance of the differences was studied using the Mann-Whitney U test for non-parametric variables. Statistical significance was regarded when p < 0.05.

## Competing interests

The author(s) declare that they have no competing interests.

## Authors' contributions

MS performed most experiments and wrote the manuscript. MB and AK helped perform experiments. DS, EH and YM participated in the study design and helped to draft the manuscript. All authors read and approved the final manuscript.
